# Mechanisms underlying the changes in acetaldehyde dehydrogenase 1 in cholangiocarcinoma

**DOI:** 10.7150/jca.86967

**Published:** 2023-09-25

**Authors:** Bai Ding, Yinghui Song, Sulai Liu, Chuang Peng, Yujing Zhang

**Affiliations:** 1 Department of Hepatobiliary Surgery, The First Affiliated Hospital of Hunan Normal University, Changsha, 410005 Hunan Province, China; 2 Central Laboratory of Hunan Provincial People's Hospital (The First Affiliated Hospital of Hunan Normal University), Changsha, 410015, China.; 3 Key Laboratory of Molecular Epidemiology of Hunan Province, School of Medicine, Hunan Normal University, Changsha, China.

**Keywords:** Cholangiocarcinoma, ALDH1, prognosis

## Abstract

Cholangiocarcinoma (CCA) is the most recurrent malignant tumor found in the biliary system. It originates from the bile duct epithelial cells characterized by easy metastasis, high intermittent rate, and poor prognosis. Acetaldehyde dehydrogenase 1 (ALDH1), a marker of cancer stem cells, the levels of which are particularly elevated in various of malignant tumors. Additionally, the increased ALDH1 levels are closely related to the degree and prognosis of malignant tumors. This study reviewed the mechanisms underlying the changes in ALDH1 levels in CCA.

## Introduction

Cholangiocarcinoma (CCA) is a common primary liver malignancy, accounting for approximately 15% of all primary liver tumors 1-3. CCA is categorized into three subtypes according to its original anatomical sites: intrahepatic CCA, hilar CCA, and distal CCA 3, 4. At present, CCA currently accounts for 3% of all gastrointestinal malignancies and 2% of cancer-related deaths 2, 3. Radical resection (R0 resection) currently provides the most effective treatment therapy for CCA. Patients who undergo complete tumor removal can have a median survival time of over 40 months, with a 25% to 40% overall survival rate after five years. On the other hand, patients who did not go through surgical resection had a much lower median survival time of only 10-12 months 3, 5, 6. Since patients with CCA are often asymptomatic in the early stages with a lack of specific tumor markers, when diagnosing, the disease is often at an advanced stage, resulting in limit treatment options and resulting in a poor prognosis 7.

Acetaldehyde dehydrogenase (ALDH) is an intracellular oxidase that alleviates intracellular acetaldehyde or drug toxicity, and also a significant cancer stem cell (CSC) marker that influences a series of important processes such as relief from ethanol or drug cytotoxicity 8-10. ALDH1, an important member of the ALDH family, is mainly categorized into ALDH1A1, ALDH1A2, ALDH1A3, and other subtypes. The ALDH1A1 coding gene is located on autosomal chromosome 9 with 13 exons, while ALDH1A2 and ALDH1A3 are located on chromosome 15 11, 12. ALDH1, an enzyme that oxidize retinol to retinoic acid (RA), is involved in cell proliferation and RA-related metabolism 13, 14. At the same time, it shows antioxidative activity and the ability to regulate osmotic pressure, participating in drug metabolism and cell differentiation. ALDH1 also serves as a marker significantly related to CSC, which can be used to extract tumor cell subsets from various cell lines and primary tumors 15. In recent years, the expression of ALDH1 has been detected in CCA, therefore, we reviewed the significance of ALDH1 expression in CCA.

## 1. Tumor-associated signaling pathways and proteins affect the expression of ALDH1

### 1.1 Common regulatory proteins for the transcription of ALDH1 in tumor cells

**Figure 1:**
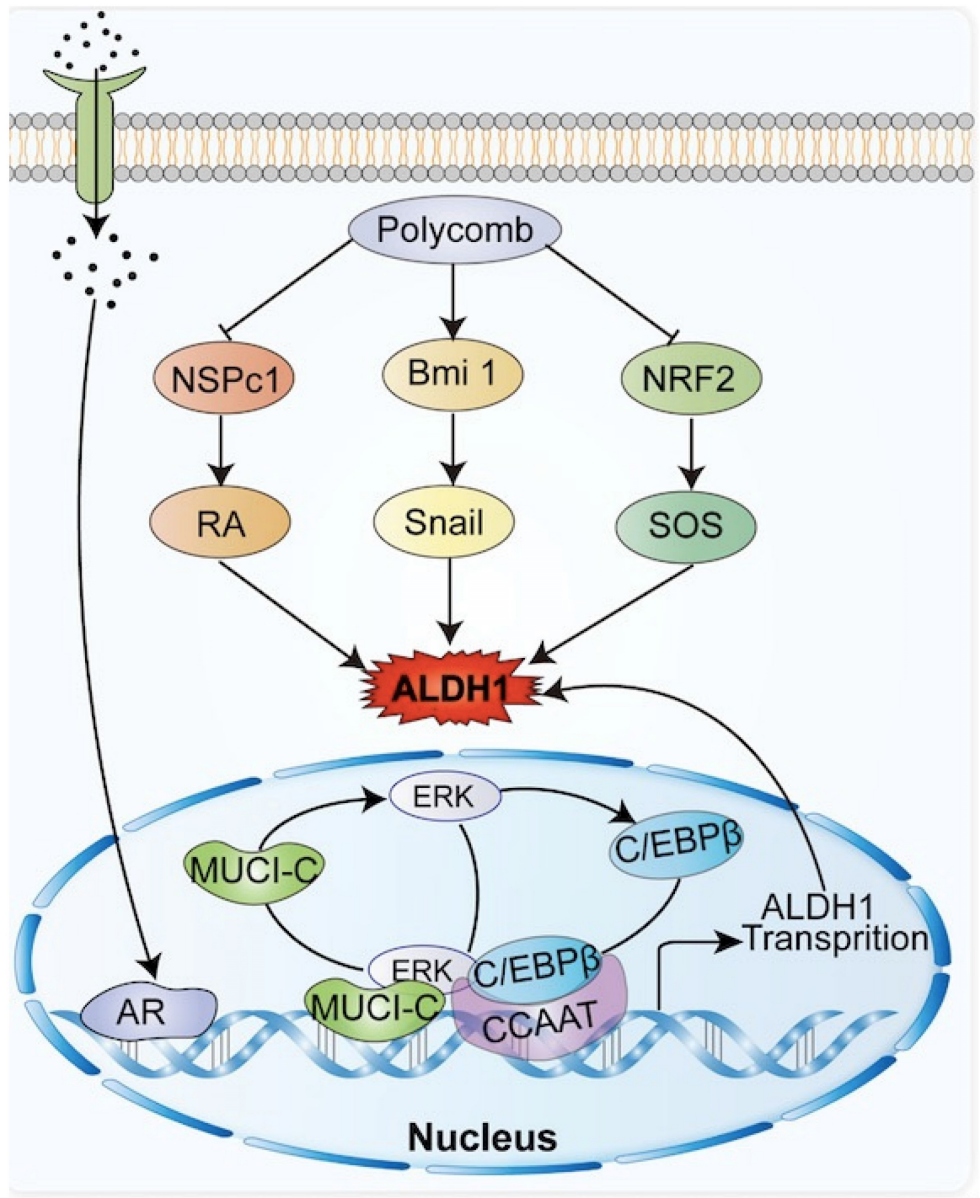
Common molecules in tumors related to ALDH1, including polycomb family genes, androgen, NRF2, etc., promote the expression of ALDH1 by interacting with ALDH1.

The ALDH1 gene promoter contains a CCAAT box (71 to 67 bp) activated by the CCAAT/enhancer binding protein β (C/EBPβ), and the binding of C/EBPβ to the promoter initiates ALDH1 expression 16, 17 In breast cancer, mucin 1 (MUCI-1) induces phosphorylation of extracellular regulated protein kinase (ERK) and activation of C/EBPβ transcription factors (Figure 1). MUCI-1, phosphorylated ERK, and activated C/EBPβ transcription factors form a complex at the ALDH1 promoter and activate ALDH1 18. Aberrant activation of the signal transducer and transcription 3 activator (STAT3) significantly promotes DNA transcription of ALDH1 19. STAT3 forms a complex with the nuclear factor kappa-light-chain-enhancer of activated B cells (NF-κB), which interferes with the interaction between C/EBPβ and DDIT3/CHOP/GADD153 to form a complex avoiding C/EBPβ inactivation thus allowing C/EBPβ-dependent ALDH1 promoter expression 20. In addition, the ALDH1 promoter contains androgen receptor (AR) binding sites. So, direct regulation of the AR pathway regulates ALDH1 expression in androgen-dependent prostate cancer cells (Figure 1). The androgen dihydrotestosterone directly upregulates ALDH1 by inducing AR transcriptional activity 21, 22. Tumors associated with AR, such as prostate cancer, breast cancer, and endometrial cancer, all show high expression levels of ALDH1 21.

### 1.2 Interactions between ALDH1 and molecules in tumors

In breast cancer, Notch signal pathway induces deacetylation and activation of ALDH1Lys-353 through mammalian Sir2 homologue (SIRT2), controls the activity of ALDH1 (Figure 1), and is related to tumorigenesis and tumor growth 13. The nuclear erythroid-associated factor 2 (NRF2) is a protein associated with erythroid and platelet development. NRF2 (Figure 1) is involved in antioxidant processes, and high levels of NRF2 interact with antioxidants and detoxifying enzymes to involve in tumor chemoresistance 23. Increased expression of NRF2-mediated antioxidant enzymes inhibits reactive oxygen species (ROS) production, and reduced ROS production serves as a negative feedback signal promoting ALDH1 expression 24. Polycomb proteins are a family of proteins, relating to CSC and tumor transformation, which could reshape chromatin and thereby express epigenetically silence genes (Figure 1). The Polycomb genes Bmi-1 and NSPc 1 can promote ALDH 1 expression 25, 26.

A high expression of ALDH1 could increase the risk of malignant tumors related to ethanol 27. Mounting evidence suggests that patients with malignant tumors exhibiting a high expression of ALDH1 have an increased propensity for metastasis and a poor prognosis 21. Other malignant tumors, such as acute lymphoblastic leukemia and prostate cancer, tumor cells exhibit higher ALDH1 activity levels and a worse tumor prognosis with increasing tumor invasiveness 28, 29. ALDH1 interacts with the common genes and signal pathway proteins in tumors. Tumor-promoting factors can upregulate the expression of ALDH1, promoting occurrence, proliferation, and metastasis of tumor cells, subsequently, enhancing tumor resistance.

## 2. Mechanism of action of ALDH1 in CSC

CSC, known as tumor-initiating or tumor-proliferating cells 30, are also the underlying cause of tumor heterogeneity. CSC are a subdivision of tumor cells with characteristics of self-renewal, high tumorigenicity, and multidirectional differentiation 31. An increasing number of studies have reported that ALDH1 shows high activity in a variety of CSC, and its expression is closely associated with tumor metastasis, drug resistance, and restenosis. In ALDH1 family, ALDH1A1 and ALDH1A3 are especially significant in CSC 32-34. ALDH1 is involved in many signaling pathways that regulate CSC, including RA and transforming growth factor-β (TGF-β) 35.

### 2.1 Enzymatic functions of ALDH1 and CSC

In the presence of ALDH1, retinaldehyde is irreversibly metabolized to RA (all-trans, 9-cis, 13-cis) 36, 37. RA is a signaling molecule that regulates the transcription of gene and is involved in the differentiation and proliferation of CSC 38. In the classical RA pathway, the RA molecule enters the nucleus and binds with the heterodimer created by nuclear retinoic acid receptor (RARα) and retinol X receptor (RXR). Histone acetylation induced by RA activation promotes downstream AKT molecule activation, upregulation of ALDH1 expression, and promotes CSC differentiation and cell proliferation 39.

ALDH1 plays a role in acetaldehyde metabolism that facilitates CSC proliferation. Ethanol is metabolized into acetaldehyde through the actions of ethanol dehydrogenase (ADH), catalase, and cytochrome P4502E1 (CYP2E1), which disrupt antioxidant defense systems and produce ROS. ROS inhibit DNA repair and methylation, forming the DNA and protein complexes that inhibit the growth of CSC 31, 33, 40. ALDH1 oxidizes toxic aldehydes while removing ROS, preventing the toxic effect of retinoids and ROS to CSC 41.

### 2.2 ALDH1 induces epithelial-mesenchymal transition associated with CSC

Epithelial-mesenchymal transition (EMT) is not a single stereotyped process, but a multistep process that involves a transition from epithelial to mesenchymal cells through an intermediate mixed state (partial EMT state, P-EMT) 42-44. While the TGF-β-induced transition from epithelial to P-EMT is reversible, the transition from P-EMT to mesenchymal cells may not be reversible 45. In the case of CCA, the TGF-β pathway induces EMT. The acquisition of mesenchymal stroma, assisted by upregulation of ALDH1 expression, is a key event in increasing tumor aggressiveness and CSC properties 46, 47. EMT allows tumors to acquire a pluripotent stem cell-like phenotype of tumor cells and to express CSC-specific transcription factors. SOX and others are involved in maintaining the multiple differentiation potential of tumors. Meanwhile, Notch1 and Wnt2 regulate CSC self-renewal 48.

CSC have been demonstrated to be crucial in tumor growth. ALDH1, as a practical marker of CSC, indicates the acquisition of tumor stemness and helps the discrimination of CSC in tumors 49.

## 3. ALDH1 in the prognosis of CCA

### 3.1 ALDH1 expression indicates a poor prognosis in CCA

In patients with CCA, high expression of ALDH1 is consistently associated with poor tumor differentiation and a poor prognosis, which agree with the findings from studies on other types of tumors 50, 51. In breast cancer, ovarian cancer, and many other tumors, high expression of CD274 (PD-L1) can activate the immunosuppressive response and lead to tumor progression 52-54. In contrast, low expression of CD274 in CCA is associated with an increase in ALDH1 activity and a reduction in the production of active aldehyde and ROS, resulting in the expression of the CSC characteristics of CCA cells. Meanwhile, the levels of the CSC markers Oct4, Sox2, and Nanog are also elevated 55. As a negative tumor regulator of CCA, CD274 downregulates the expression of the CSC marker ALDH1 and decreases the production of ROS and active aldehydes.

LIN28B is a type of RNA-binding protein 56. The activity and expression level of the CSC marker ALDH1 are especially increased in CCA cell lines overexpressing LIN28B. Furthermore, LIN28B overexpression in CCA cells activates the carcinogenic signaling pathway and leads to increased STAT3 expression 57. STAT3 has the ability to participate in ALDH1 transcription and reduce the sensitivity of chemotherapeutic drugs. The LIN28B/STAT3/ALDH1 pathway play a complicated role in bile duct cell proliferation and differentiation differentiation and is associated with the occurrence of CCA. These results show that ALDH1 is a CSC marker of CCA, and a prognostic factor for CCA 57.

### 3.2 Signal pathways related to ALDH1 and CCA

#### 3.2.1 ALDH1 and the classical tumor activation pathway

**Figure 2:**
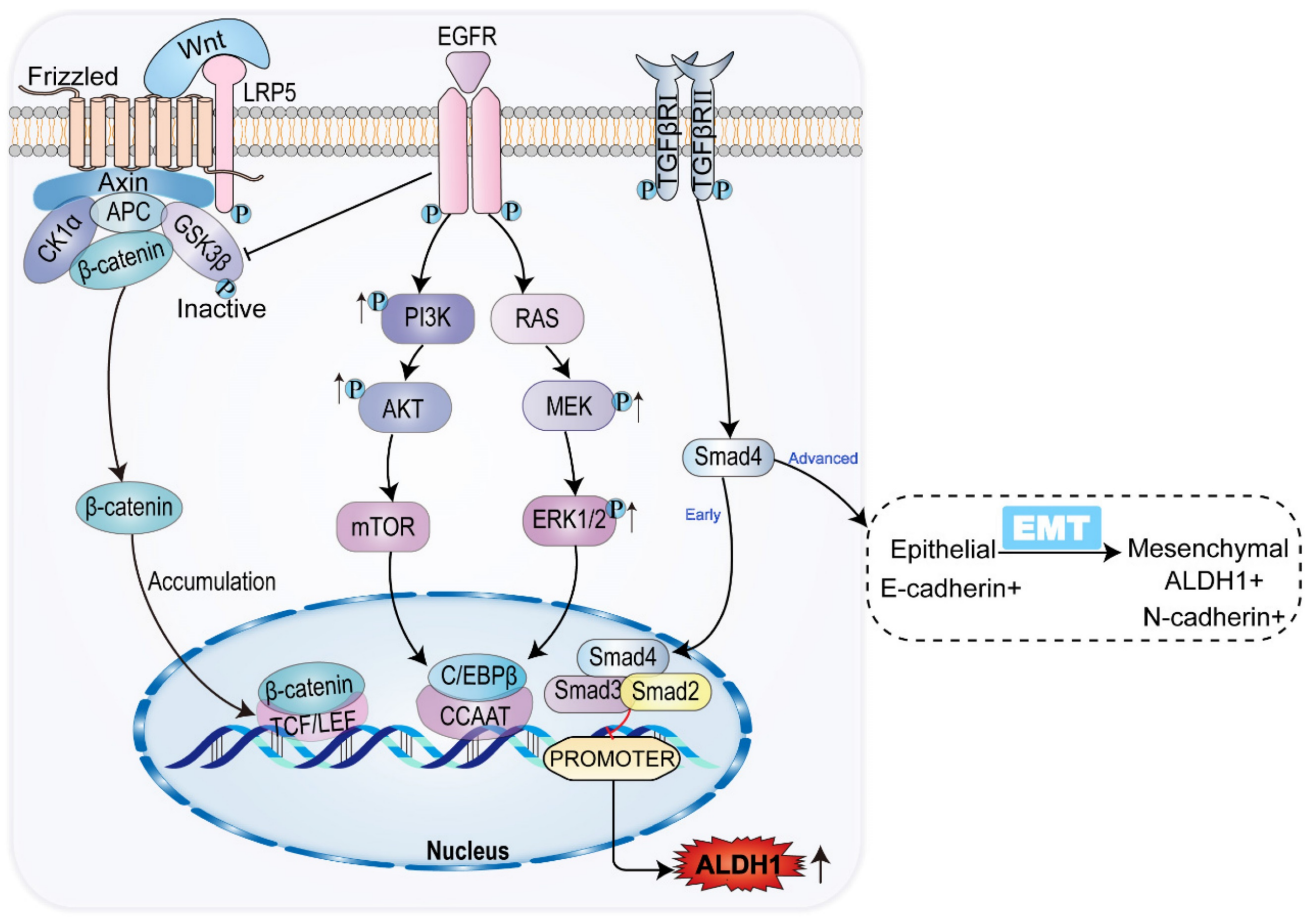
Common signaling pathways and ALDH1 expression in CCA. In CCA, WNT/β-catenin signaling pathway promotes ALDH1 expression by binding to transcriptional regulatory factor TCF, while classical tumor signaling pathways PI3K/AKT/mTOR and RAS/MEK/ERK promote ALDH1 expression by promoting transcriptional enhancer CCAAT. TGF-β/Smad signaling pathway has the opposite effect on the expression of ALDH1 in the early and advanced stages of the tumor, inhibiting the expression of ALDH1 in the early stage and vice versa in the late stage.

Interleukin-6 (IL-6), vascular endothelial growth factor (VEGF), epidermal growth factor (EGF), and hepatic liver growth factor (HGF) participate in classical oncogenic pathways, including the RAS-MAPK-ERK-MTOR and PI3K-AKT-MTOR pathways (Figure 2). Expression of ALDH1 can be upregulated through the classical carcinogenic pathway 58. The mammalian target of rapamycin (mTOR), a 289-kDa serine/threonine protein kinase, regulates cell proliferation, survival, and angiogenesis 12, 59. IL-6 and other agents can increase the expression of granulin precursor proteins, which activate the AKT-mTOR pathway of cell mitosis, cell migration, and angiogenesis. Meanwhile, mTOR activates downstream ALDH1A1 expression 47, 60.

IL-6 and others activate p38MAPK, a group of protein kinases responsible for cell differentiation and proliferation, promoting ALDH1 expression and leading to reduced expression of p21, a mediator of cell senescence, as well as to promotion of mitosis, and enhancement of cell proliferation 61. MAPK can enable ERK phosphorylation and activation, which activates mTOR. Activation of the RAS-MAPK-ERK-MTOR pathway subsequently activates downstream ALDH1A3 12. The expression of mTOR is common in gallbladder cancer and CCA, suggesting a tumor prognosis. Inhibition of mTOR exhibits antitumor effects in gallbladder cancer and CCA 62. Additionally, mTOR can be activated by phosphorylation of C/EBPβ2. Activation and proliferation of C/EBPβ2 upregulates the expression of ALDH1 by recognizing and binding to the ALDH1 promoter located in the CCAAT box of the cis-acting element 59. ALDH1 expression is upregulated in the classical tumor activation pathway, which is closely related to the growth and progression of CCA, indicating a poor prognosis for CCA.

#### 3.2.2 ALDH1 and TGF-β-SMAD pathway

TGF-β plays a dual role in cancer. In addition to controlling CCA progression by inducing apoptosis, TDF-β can also increase the progression of CCA by promoting EMT, tumor migration and invasion, and suppressing the immune system 63.

In the early stage of CCA, TGF-β inhibits the expression of ALDH1 via SMAD, while the TGF-β signaling pathway is associated with a decrease in tumor-initiating cells in CCA. The decreased expression of ALDH1 suggests that tumor activation is regulated in CCA 64, 65. However, in advanced CCA, activation of the TGF-β signaling pathway can contribute to tumor growth, with tumor progression 15, 66. The percentage of ALDH1-positive cells significantly increases in CCA cells treated with TGF-β. Meanwhile, ALDH1-positive cells exhibit stem cell characteristics and EMT features 47, 67-69. TGF-β induces the level and activity of SMAD, promoting EMT by inhibiting the transcription of E-calmodulin, the epithelial morphological marker 70. EMT is the key to acquiring CSC properties 71. In the TGF-β pathway, ALDH1 expression is upregulated and the invasive activity of CCA is elevated 46. The results of immunohistochemical analysis showed that both TGF-β and ALDH1 expression were enhanced in advanced CCA (Figure 2). Consequently, TGF-β and ALDH1 could be independent prognostic factors in CCA, where the upregulation of ALDH1 and TGF-β expression indicated shortened overall survival of patients 46. ALDH1 acts as a prognostic marker for CCA, indicating a poor prognosis of CCA in both in the early and late stages of the tumor.

#### 3.2.3 ALDH1 and WNT-β-catenin pathway

When the bile duct epithelium is damaged, inflammatory macrophages produce WNT ligands, which usually play an epithelial repair role 72. Macrophages upregulate the expression and transcription of WNT7b and WNT10a, inhibiting the degradation of intracellular β-catenin by binding to the FZD receptor and its co-receptor LRP5/LRP6 on bile duct cells, thus leading to β-catenin accumulation 73. In the nucleus family, β-catenin interacts with the TCF/LEF transcription factor, causing the expression of ALDH1. This leads to increased cell viability and resistance to apoptosis 74. SOX17, an oncogene, encodes a protein that can forms a complex with other proteins and act as a transcriptional regulator. It antagonizes the WNT-β-catenin signaling pathway, negatively regulates the expression of ALDH1, regulates cholangiocyte differentiation, and acts as a tumor suppressor in CCA (Figure 2). Studies have shown that the SOX17 promoter is highly methylated, resulting in decreased expression of SOX17. This reduces the inhibition of the WNT-β-catenin pathway, leading to upregulation of ALDH1 expression, and worsening the prognosis of CCA 75, 76.

### 3.3 The epigenetics of ALDH1 is associated with CCA

**Figure 3:**
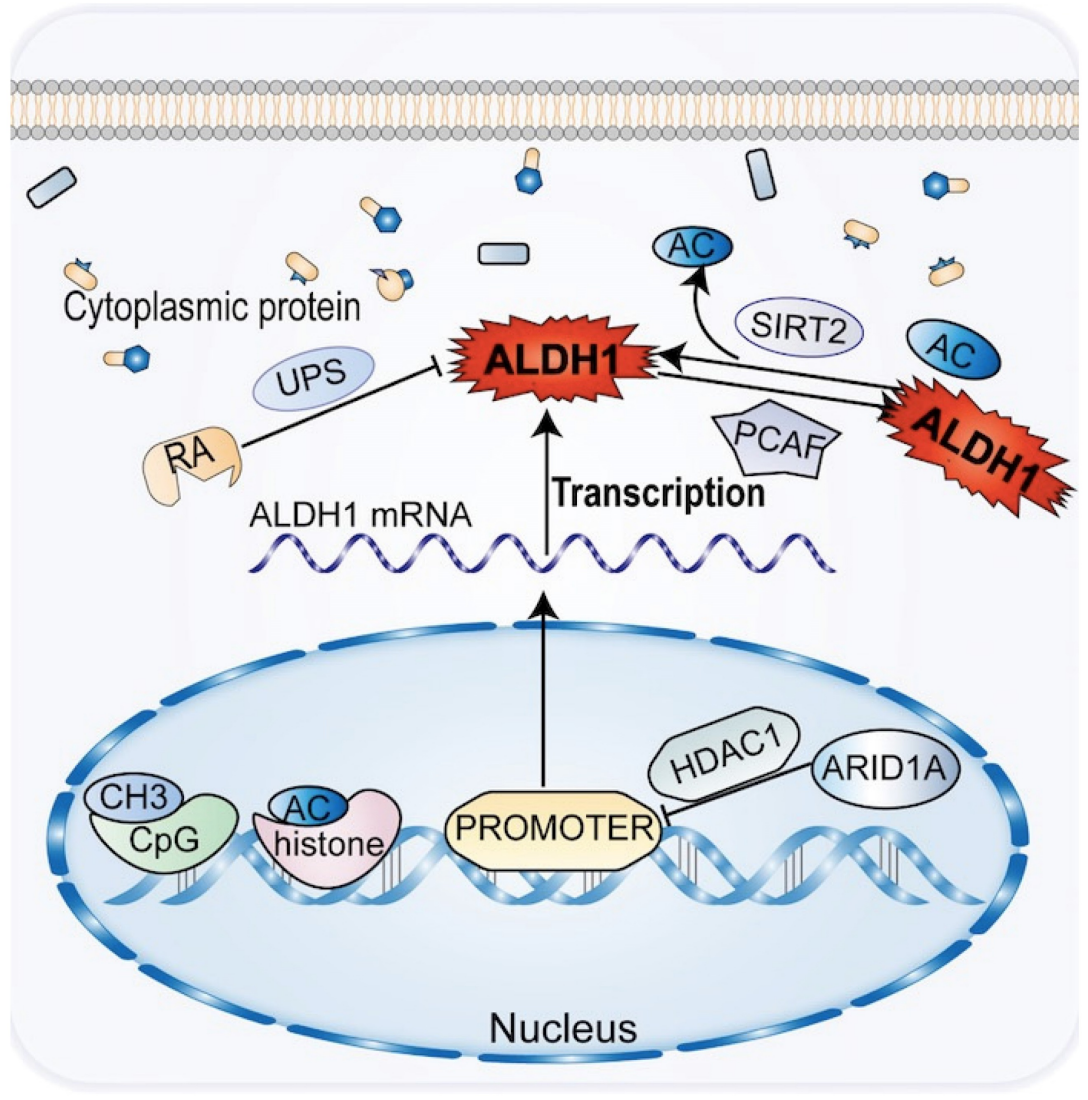
The common genetic epigenetic regulation of ALDH1 in CCA is that ALDH1 expression is down-regulated by methylation and acetylation before transcription, and ALDH1 protein activity is inhibited by ubiquitin and acetylation after transcriptional translation.

Chemical modifications of epigenetic and post-translational proteins can impact the expression and activity of ALDH1 in CCA. Epigenetics plays an important role in the development and progression of CCA, affecting the tumor phenotype in the absence of changes in the DNA sequence 77. Histone acetylation modifications and methylation of CpG islands regulate the transcriptional promoters of ALDH1A1 and ALDH1A3 to downregulate the corresponding ALDH1 12. Genes such as ALDH1A1, SPP1, and CD81 have been shown to be upregulated in ARID1A-deficient CCA cells, with the most significant changes in ALDH1A1 78. By reducing histone H3K27 acetylation, ARID1A introduces histone deacetylase into the promoter region of ALDH1A1, suppressing the expression of ALDH1A1 in CCA cells, thereby acting as a tumor suppressor of CCA (Figure 3). In CCA patients, deletion of ARID1A and upregulation of ALDH1A1 are independent prognostic factors 78.

Following post-translational chemical modification in CCA, ALDH1 can oxidize retinoids to form RA and its analogs (Figure 3). RA and its analogs, particularly tran-sretinoic acid, can reduce ALDH1 activity through the ubiquitin protease system without altering ALDH1 mRNA levels. However, RA may be altered by protein chemical modification of ALDH1 activity 79, 80. Acetylation of the lysine 353 is another post-translational modification of ALDH1. ALDH1 activity decreases by acetylation of lysine 353, a process activated by acetyltransferase P300/CBP-associated factor (PCAF) 81, 82. Notch signaling induces high ALDH1 activity in CCA. This process increases the expression of deacetylase sirtuin2 (SIRT2), which allows the elimination of ALDH1 acetylation 13, 81. A reduction in ALDH1 activity leads to an increase in ROS and reactive aldehydes in CSC and promotes CSC apoptosis in CCA.

## 4. Relationship between ALDH1 and chemotherapy resistance in CCA

The GC and GEMOX regimens are standard first-line chemotherapy treatment for CCA 5. Gemcitabine (GEM) plays a critical role in various first-line chemotherapy treatment. The occurrence of GEM chemotherapy resistance often predicts a poor prognosis in CCA. Elevated ALDH1 activity has been connected to the increased drug resistance and metastasis in a range of cancers 83-86. Furthermore, in patients with advanced CCA, ALDH1 has been shown to play a crucial role in malignant behavior and resistance to GEM 87.

### 4.1 Mechanism of resistance to GEM through ALDH1 expression in CCA

Intrahepatic cholangiocarcinoma cell lines HuCCT1 and SNU1079 showed high expression of ALDH1, primarily by ALDH1A3 87. ALDH1 is a universal CSC marker, and most studies have reported that the long-term dormancy of CSC confers resistance to most drugs in fast-growing cancer cells. A high expression of ALDH1 is also closely related to EMT and promotes the acquisition of resistance to GEM. The occurrence of EMT is often associated with drug resistance in CCA cells 88. ALDH1 upregulates and induces resistance mainly by increasing the population of CSC and promoting the appearance of EMT to most chemotherapeutic drugs administered for CCA 89.

**Figure 4:**
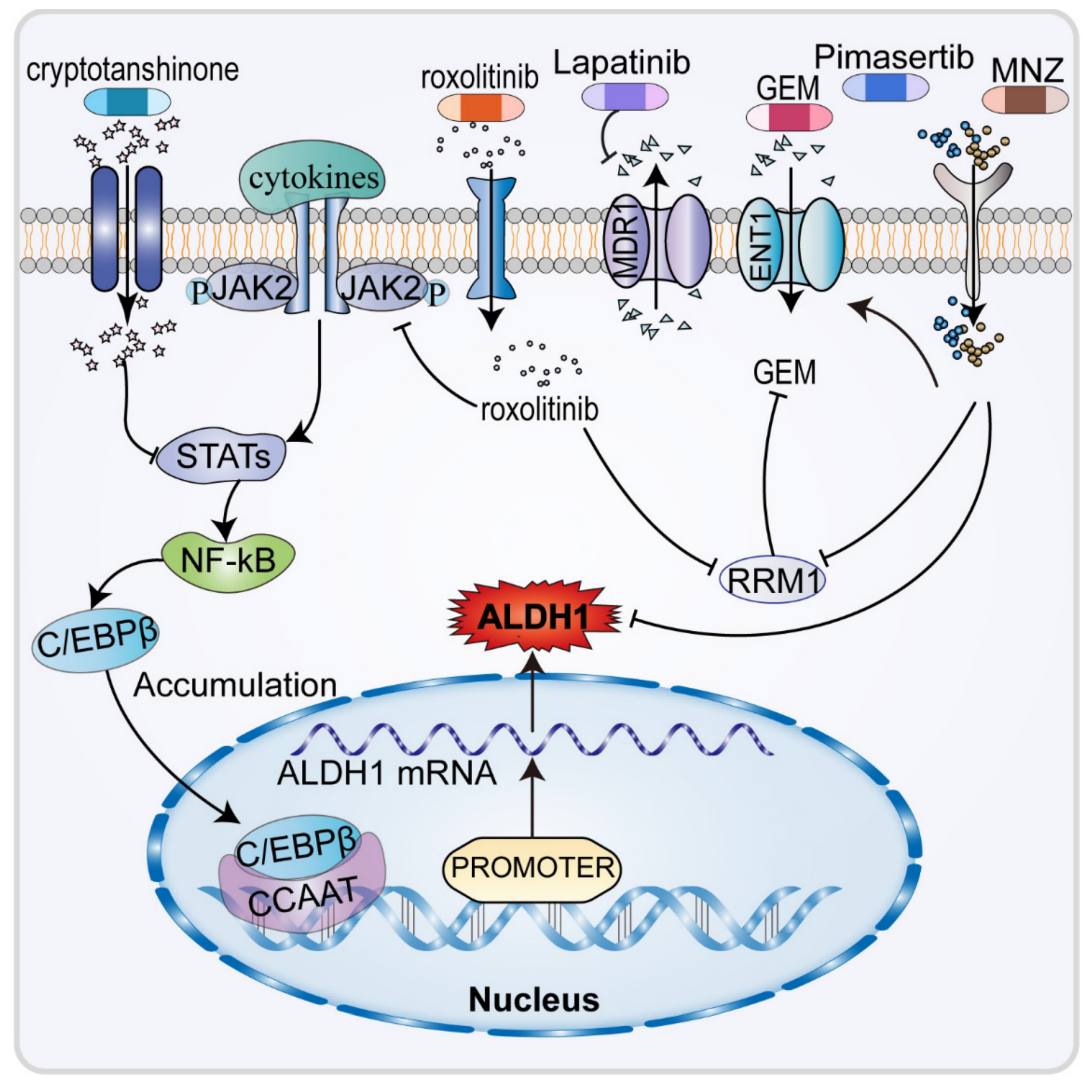
The interaction between ALDH1 and gemcitabine, in CCA, the expression of ALDH1 is often accompanied by gemcitabine resistance, and the up-regulation of ALDH1 is accompanied by an increase in the exclusion of gemcitabine and a decrease in gemcitabine entry. Related chemicals restore the sensitivity of gemcitabine to cholangiocarcinoma by inhibiting the expression of ALDH1.

In human CCA cell lines with high expression of ALDH1, CCA stem cells are resistant to GEM but not to cisplatin. The reason cannot be explained by the long-term dormancy of CSC as both drugs inhibit fast-growing cancer cells. GEM is a difluoro nucleoside antimetabolites anticancer drug that inhibits cell replication 90, by functioning as a water-soluble analogue of deoxycytidine and competitively inhibiting ribonucleotide reductase 1 (RRM1) 91. It acts on pyrimidine deoxyribonucleic acid and requires conversion to gemcitabine triphosphate before acting with DNA to inhibit DNA synthesis and induce cell apoptosis 92. The expression of RRM1, which is the molecular target of ALDH1A3, increased significantly in HuCCT1 and SNU1079 cells treated with high doses of GEM (Figure 4). RRM1 is a polymerase that converts ribonucleotides into deoxyribonucleosides and plays a critical role in DNA polymerization and repair 93. RRM1 is also crucial in resistance to gemcitabine and can competitively inhibit the role of GEM 94. This study demonstrated that RRM1 levels decreased, resulting in higher GEM response rates in patients with lower expression levels of ALDH1A3 who received GEM chemotherapy for CCA. The correlation between ALDH1A3 expression levels and chemotherapy responses was verified in 31 patients 87.

### 4.2 ALDH1 participates in the mechanisms underlying anti-GEM resistance

The therapeutic options for advanced chemotherapy refractory CCA are limited, highlighting the need to identify of new effective therapeutic agents (Figure 4). It has been found that the amatuximab and dasatinib can inhibit adhesion of cancer cells to the peritoneum and suppress the stemness and viability of cancer cells, reducing ALDH1 expression and improving tumor sensitivity to GEM 40, 95. Infigratinib downregulates ALDH1 expression in CCA cells by inhibiting FGFR/AKT/MTOR and FGFR/STAT3, which also suppresses EMT, to increase the antitumor effect of GEM 96. In patients with advanced CCA, ALDH1 plays an important role in malignant behavior and resistance to GEM 87. As an inhibitor of Janus kinase 2 (JAK2), the targeted drug ruxolitinib inhibits downstream signal transduction and the expression of STAT1/3, downregulating the expression of ALDH1, thereby increasing tumor sensitivity to GEM 19. Ruxolitinib has also been found to significantly inhibits the migratory capacity of CCA cells. Fraxetin and cryptotanshinone, the anti-dysentery drugs, can enhance the antitumor effect of GEM by inhibiting STAT3 which results in the downregulation of ALDH1 expression 97, 98.

Previous studies (Figure 4) have shown that ENT1 and RRM1 are key factors in the cytotoxicity of GEM 99-101. ENT1 is a membrane transporter protein that promotes the efficient penetration of GEM into cells 93. Upregulation of MDR1 within the ABC transporter protein family, responsible for drug efflux, lowers the concentration of many drugs, including GEM 102, 103. In CCA cells, pimasertib 104 and metronidazole (MNZ), the MEK inhibitors, decrease ALDH1 activity, increase GEM sensitivity in CCA cells by increasing equilibrium nucleoside transporter protein 1 (ENT1), thus decreasing RRM1 99. Additionally, MNZ can decrease EMT by decreasing ALDH1 activity and decreasing tumor cell stemness, which ultimately lowers invasiveness and resistance to GEM 46, 105. In CCA cells, hydroxyurea could be acted as a RRM inhibitor 106. Moreover, lapatinib and emodin increased GEM sensitivity by inhibiting MDR, accompanied by decreased regulation of ALDH1 107, 108. ALDH1 is closely related to chemoresistance in CCA, thus opening new paths for the treatment of chemo-resistant CCA.

## 5. Conclusions

As one of the markers of tumor stem cells, ALDH1 is expressed in a variety of malignant tumors relating to the clinical prognosis of patients. Alteration of ALDH1 transcription or activity may be caused by common signal pathways and molecules like WNT, TGF-β, and STAT present in tumors. The enzymatic functions of ALDH1 can confer and preserve the characteristics of tumor CSC. Signaling pathways associated with ALDH1 can promote the completion of EMT as well as the acquisition of tumor dryness.

A comprehensive and current literature review revealed that ALDH1 plays an important role in the degree of malignancy, efficacy evaluation, and prognosis of CCA. On the other hand, the typical signaling pathways in CCA may impact the expression of ALDH1. In CCA, ALDH1 participates in the TGF-β/SMAD and WNT/β-catenin pathway, and its expression level is positively correlated with the tumor malignancy of CCA. Epigenetics regulates the expression of ALDH1 before and after translation and transcription, contributing to changes in the degree of malignancy of CCA.

ALDH1 is closely associated with chemotherapy resistance and EMT in CCA. GEM is the foundational drug for CCA chemotherapy, and the resistance to this drug is linked to the upregulation of ALDH1. Although new drugs have been demonstrated to inhibit the expression of ALDH1 and facilitate the recovery of chemosensitivity in CCA, the specific mechanisms underlying the expression of ALDH1 in CCA remain to be further discovered. Resistance to tumor radiation therapy, immunotherapy, and targeted therapy is not thought to be linked with the expression of ALDH1. A thorough understanding the function of ALDH1 is highly beneficial for the treatment and prognosis of CCA. Research on ALDH1 will also generate novel approaches for the treatment of CCA.
